# Chylothorax from Bilateral Primary Burkitt's Lymphoma of the Ovaries: A Case Report

**DOI:** 10.1155/2012/635121

**Published:** 2012-07-18

**Authors:** A. C. Etonyeaku, O. O. Akinsanya, O. Ariyibi, A. J. Aiyeyemi

**Affiliations:** ^1^Surgery Department, Federal Medical Centre, Owo, Ondo State, Nigeria; ^2^Department of Surgery, Obafemi Awolowo University, Ile-Ife, Osun State, Nigeria; ^3^Obstetrics & Gynaecology Department, Federal Medical Centre, Owo, Ondo State, Nigeria; ^4^Histopathology Department, Federal Medical Centre, Owo, Ondo State, Nigeria

## Abstract

We present a case of bilateral ovarian Burkitt's lymphoma is an 18-year old. Diagnosis was made at histology of specimens obtained after an exploratory (diagnostic) laparotomy. Disease was advanced at presentation and complicated with both chylothorax and chylous ascites. Response to chemotherapy though dramatic was short lived. This underscores the need for high index of suspicion amongst clinicians. The availability of affordable less traumatic diagnostic procedures like laparoscopy and computerized tomography scans with facilities for guided biopsies in resource-poor settings can fast track diagnosis and hence treatment.

## 1. Introduction 

Burkitt's lymphoma (BL) is common in areas of high malaria endemicity [1], in children and young adults [2] and is strongly associated with the Epstein-Barr virus infection. In males the jaws bones are mainly affected while in females the ovaries are involved. The lymph nodes are rarely involved. Diagnosis of intra-abdominal Burkitt's Lymphoma is often delayed, as symptoms could be vague and non-specific. the may explain why patients often present with advanced disease. Attention is paid more durring clinical, radiological and laboratory assessment for acute abdomen, unexplained weight loss associated with abdominal distension. In the tropics abdominal tuberculosis may mimic the disease [3], hence the need to properly evaluate with histology of specimen. BL responds well to chemotherapy; prognosis is poor in advanced disease state. One of the documented causes of chylous ascites and chylothorax is lymphoma [4]. In presence of either or both of these a high index of suspicion is advised. 

We present a case of primary ovarian Burkitt's lymphoma involving both ovaries and complicated with chylous ascites and chylothorax. We advise that, in the presence of bilateral ovarian tumour, secondary amenorrhoea, chylothorax, and chylous ascites BL should be excluded. 

## 2. Case Presentation 

Miss AH was an 18-year old unmarried school leaver who had a four-week history of progressive abdominal pain and lower abdominal swelling. Pain was insidious, dull in character and was worse over a lower abdominal swelling that was noticed about the same time. There was no cough, nor history of contact with someone with chronic cough. There was occasional difficulty with breathing. There was no fever or drenching night sweats. There was weight loss which was attributed to anorexia, early satiety with occasional vomiting of recently ingested meals. There was no swelling in any part of her body. There was no change in bowel habit or lower urinary tract symptoms. Other systems reviewed were essentially normal. Lately menses had been irregular. She was not sexually active and not sure of date of her last menstrual flow. Family and social and drug history were not significant. 

At presentation she was chronically ill, in painful distress, afebrile, not pale, anicteric, and she had no significant peripheral lymphadenopathy and no peripheral oedema. Vital signs were within normal limit and chest was clinically clear. There was a tender suprapubic mass about 22 weeks gestational size that was slightly mobile transversely. Bowel sounds were normal. The hymen was intact. Digital rectal examination revealed a normal rectal mucosa with a bulging pouch of Douglas but no Blummer's shelves. 

A provisional diagnosis of intra-abdominal malignancy of probable ovarian origin was made. 

**Figure 1:**
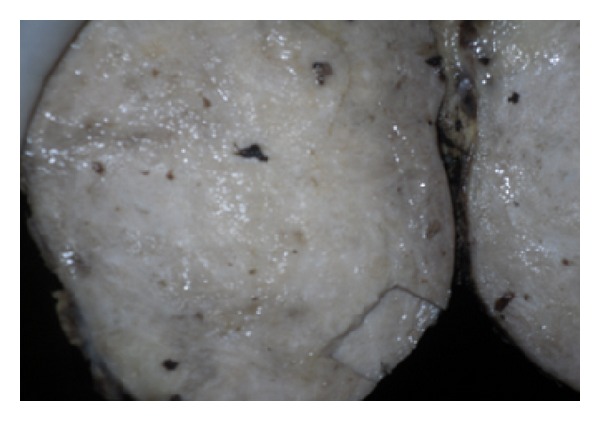
Homogenous greyish-white surface.

**Figure 2:**
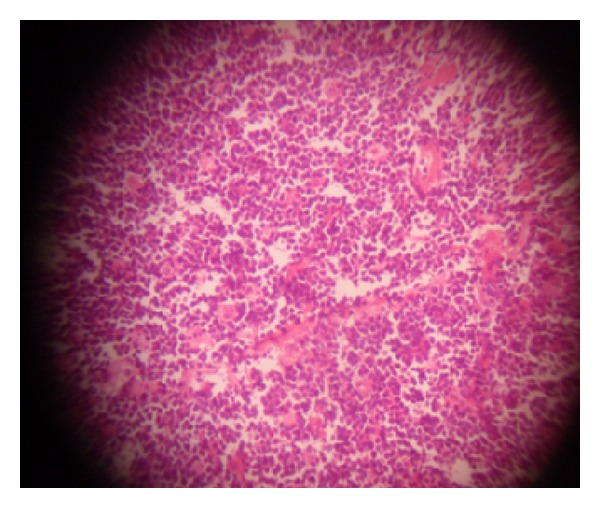
Uniform atypical lymphoid cells with starry sky appearance.

**Figure 3:**
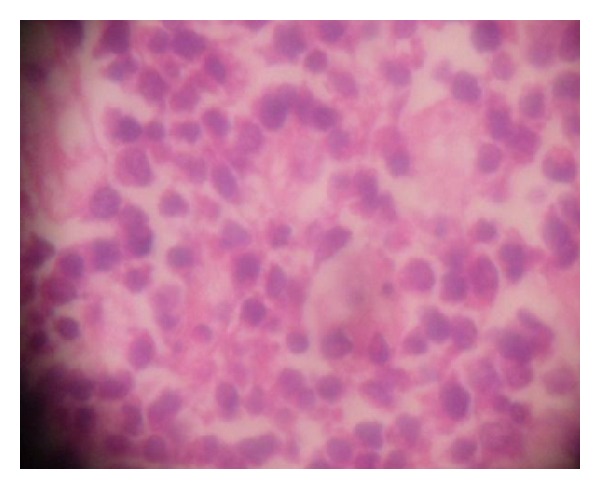
×100 magnification.

Full blood count was normal with relative lymphocytosis (54%). Serum electrolytes and urea were within normal limits. Abdominal ultrasonography suggested intra abdominal masses of probable ovarian origin. Computerized tomography scan and laparoscopy were considered but not done because the services were not available and patient could not afford them in centres where they are available. She was HIV I and II negative on the determine kit. She had exploratory laparotomy. Intra-op findings were of copious milky blood-stained intra peritoneal collection, bilateral ovarian masses with induration of tubes, broad ligaments, and the greater omentum. The diaphragm, liver, splenic surfaces and guts were grossly normal. A left salpingoophorectomy and wedge biopsy of the omentum and right ovary were done. These specimens (Figure 1) were sent for histology while the ascetic fluid was tested for acid/alcohol fast bacilli (AAFB) test. The AAFB test was negative. She had empirical treatment for disseminated tuberculosis with rifampicin, isoniazide, ethambutol, and pyrazinamide. On the14th day after operation she had an underwater seal-closed right thoracostomy tube drain for massive chylothorax. Histology of the surgical specimens indicated Burkitt's lymphoma (Figures 1, 2, and 3). The peripheral blood film showed normal white cell count with relative lymphocytosis, and bone marrow biopsy was not indicative of involvement. The anti-Koch's therapy was discontinued. She had two courses of combination therapy with cyclophosphamide, vincristine, and methotrexate. Response was remarkable after the first course, but patient died after the second course of therapy probably from tumour lysis syndrome or chemotoxicity; despite the use of allopurinol prophylaxis. post-mortem study to ascertain cause of death was advised but the deceased family declined the study. 

## 3. Discussion 

Ovarian malignancies are common and histological type varies with age and may either be primary or secondary. BL is a common extranodal disease occurring most often in children and immune-compromised individuals [5]; it has been reported to be the most common type of ovarian tumour in Nigeria patients less than 20 years old [6]. Three distinct forms of BL have been described, namely, endemic, sporadic (nonendemic), and immune deficiency related [7]. The nonendemic type is associated with abdominal disease (including the gonads), breast, bone marrow, and central nervous system. Primary ovarian Burkitts involving both ovaries is rare accounting for about 0.5–1% of all ovarian lymphomas [8, 9]. Extragonadal involvement occurs early affecting the omentum, fallopian tubes, and lymph node [10] as seen in our patient. Menstrual irregularity is the most common menstrual disorder and was noted in our patient; however normal menses have been reported in some women with bilateral disease [11]. Clinical features may mimic other causes of acute abdomen or gynaecological emergencies [12]. The diagnosis of ovarian BL is usually made postoperatively on histology of operative specimen as no specific imaging feature can differentiate it from other types of ovarian neoplasm [13]. There were no facility for assay of tumour markers such as human chorionic gonadotropin (hCG), alpha fetoprotein (AFP), or carbohydrate antigen 125 (CA-125). Preoperatively, these would have helped exclude other forms of ovarian tumour. The nonavailability of frozen section services, diagnostic laparoscopy, and the two-week delay before diagnosis challenge in resource poor settings like ours. Immunohistochemistry to affirm the diagnosis was also not available. These would account for the hopeless trail of antituberculosis therapy. BL responds well to chemotherapy, and various regimens abound. At surgery she had salpingo-oophorectomy in an attempt to reduce the tumour bulk, alleviate pain, preparatory to adjuvant chemoradiation. At this stage the likelihood of diagnosis of lymphoma was not entertained. She had chemotherapy with satisfactory response after first course. The rapid deterioration of the patient may not be unconnected with natural history of the disease and toxicity of the drugs. Interval from first symptom to death was 2 months, while the tumour doubling time is 24 hours. The international prognostic index [14] (IPI) was also not favourable (though the serum lactase dehydrogenase (LDH) levels were not done). Obstruction of the lymphatic channels from extrinsic compression could result in chylous ascites and chylothorax if the serosal or pleural lymphatics dilate and leak. Again the patient had both contributing to her poor prognosis. Allopurinol is useful in preserving renal function in hyperuricemic state from tumour lysis syndrome. In advanced disease state, death could occur despite use of allopurinol perhaps from cardio-pulmonary failure which may be part of a multiple organ dysfunction state involving acute renal failure.

## 4. Conclusion 

Primary ovarian BL is rare in the general population. It tends to be relatively common in children and adolescents; it is rare in young adults. Diagnosis is often made late: sometimes postoperatively for acute abdominal states. Treatment is predominantly by combination chemotherapy. Response is good for early disease. Precaution is needed to safeguard patient from toxicity of agents and multiple organ failure from massive tumour lysis.
